# Synergism Effect of the Essential Oil from* Ocimum basilicum* var. Maria Bonita and Its Major Components with Fluconazole and Its Influence on Ergosterol Biosynthesis

**DOI:** 10.1155/2016/5647182

**Published:** 2016-05-05

**Authors:** Nathalia N. R. Cardoso, Celuta S. Alviano, Arie F. Blank, Maria Teresa V. Romanos, Beatriz B. Fonseca, Sonia Rozental, Igor A. Rodrigues, Daniela S. Alviano

**Affiliations:** ^1^Departamento de Microbiologia Geral, Instituto de Microbiologia Paulo de Góes, Universidade Federal do Rio de Janeiro, 21941-902 Rio de Janeiro, RJ, Brazil; ^2^Programa de Pós-Graduação em Biotecnologia Vegetal (PBV), Decania, CCS, Universidade Federal do Rio de Janeiro, 21941-902 Rio de Janeiro, RJ, Brazil; ^3^Departamento de Engenharia Agronômica, Universidade Federal de Sergipe, 49100-000 São Cristóvão, SE, Brazil; ^4^Instituto de Biofísica Carlos Chagas Filho, Universidade Federal do Rio de Janeiro, 21941-902 Rio de Janeiro, RJ, Brazil; ^5^Departamento de Produtos Naturais e Alimentos, Faculdade de Farmácia, Universidade Federal do Rio de Janeiro, 21941-902 Rio de Janeiro, RJ, Brazil

## Abstract

The aim of this study was to evaluate the activity of the EO and its major components of* Ocimum basilicum* var. Maria Bonita, a genetically improved cultivar, against the fluconazole sensitive and resistant strains of* Candida albicans* and* Cryptococcus neoformans*. Geraniol presented better results than the EO, with a low MIC (76 *μ*g/mL against* C. neoformans* and 152 *μ*g/mL against both* Candida* strains). The combination of EO, linalool, or geraniol with fluconazole enhanced their antifungal activity, especially against the resistant strain (MIC reduced to 156, 197, and 38 *μ*g/mL, resp.). The ergosterol assay showed that subinhibitory concentrations of the substances were able to reduce the amount of sterol extracted. The substances tested were able to reduce the capsule size which suggests they have an important mechanism of action. Transmission electron microscopy demonstrated cell wall destruction of* C. neoformans* after treatment with subinhibitory concentrations. In* C. albicans* ultrastructure alterations such as irregularities in the membrane, presence of vesicles, and cell wall thickening were observed. The biofilm formation was inhibited in both* C. albicans* strains at MIC and twice MIC. These results provide further support for the use of* O. basilicum* EO and its major components as a potential source of antifungal agents.

## 1. Introduction

Candidiasis is a fungal disease that affects a large number of individuals. Skin or mucous membranes are the most common sites of infection, especially the mouth and vagina [1]. However, the disease may evolve from a superficial infection to systemic infection, especially in immunocompromised individuals [2, 3]. The most frequent etiological agent of candidiasis is* Candida albicans*. This pathogen is an opportunistic fungus able to form biofilms, which are of major clinical concern. Moreover, fungi communities structured in extracellular polysaccharide matrices possess increased resistance to antifungal therapy [3, 4].

*C. neoformans *is responsible for cryptococcosis, a disease affecting the central nervous system and having high levels of mortality.* C. neoformans* has several well-characterized virulence factors such as capsular polysaccharide, sialic acids, and the production of mannitol and melanin. The former is considered the most important virulence factor of* C. neoformans* and therefore the most studied. The capsule contributes to the microorganism virulence through multiple mechanisms, including antiphagocytic properties and adverse effects on the host immune system [5, 6].

Despite current antifungal therapies, cryptococcal and candidal infections show unpleasant high mortality rates in immunocompromised individuals. A study conducted by Park et al. [7] revealed that cryptococcal meningitis was responsible for 624,700 deaths after 3 months of infection. A previous case-control study indicated that mortality attributable to invasive* Candida* infection was about 19–24% among hospitalized patients [8]. Factors that contribute to this scenario include a limited antifungal arsenal, diverse side effects, and drug resistant strains [1].

Essential oils (EOs) and plant extracts are commonly used in traditional medicine by indigenous populations worldwide [9, 10]. EOs are complex mixtures of volatile (terpenes, aliphatic aldehydes, alcohols, and esters) and nonvolatile components (hydrocarbons, fatty acids, sterols, carotenoids, waxes, coumarins, and flavonoids) produced by aromatic plants as secondary metabolites [11]. Various studies have attributed different biological activities to EOs such as antiviral, antigiardial, antispasmodic, carminative, analgesic, healing, expectorant, antiseptic, respiratory tract, and anti-inflammatory activities [12–16].

*Ocimum basilicum* (Lamiaceae) is found in tropical and subtropical regions of Asia, Africa, Central America, and South America [17]. Popularly known as basil,* O. basilicum* is a culinary and medicinal herb widely used in folk medicine to combat headaches, coughs, intestinal worms, and kidney disorders and as an antispasmodic agent [18]. The antimicrobial activity of basil EO has been reported to be predominantly associated with its major components, linalool and methyl chavicol [19]. The antimicrobial activity of these components and other monoterpenes has been reported in the literature against various microorganisms, including protozoans, bacteria, and fungi [20–23]. The purpose of this study was to investigate the antifungal activity of the essential oil and its major components (linalool and geraniol) from a genetically improved* O. basilicum* cultivar (*O. basilicum* var. Maria Bonita) against* C. neoformans* and fluconazole (FLC) sensitive and resistant* C. albicans* strains.

## 2. Material and Methods

### 2.1. Chemicals

Fluconazole (FLC), resazurin, 2,3-bis(2-methoxy-4-nitro-5sulfofenil)-5-[(phenylamino)carbonyl]-2H-tetrazolium hydroxide (XTT), 3-(4,5-dimethylthiazol-2-yl)-2,5-diphenyltetrazolium bromide (MTT), linalool (97%), and geraniol (98%) standards were obtained from Sigma-Aldrich (USA) and stored according to the supplier's instructions. All solvents used were of spectroscopic grade from Tedia (Fairfield, OH, USA).

### 2.2. Plant Material and Essential Oil

*Ocimum basilicum* L. var. “Maria Bonita” is a genetically improved cultivar with a high content of linalool.* O. basilicum* EO was provided by the Federal University of Sergipe, where a voucher specimen was deposited (register number 13162). EO was extracted from the leaves by hydrodistillation using a Clevenger apparatus. The chemical composition of the EO was analyzed by a GC-MS and previously published by Da Costa et al. [24] and linalool was identified as the major component of the EO (75.22%), followed by geraniol (14.66%).

### 2.3. Microorganisms

*Cryptococcus neoformans* T-444 serotype A was provided by Universidade Federal de São Paulo (UNIFESP).* C. albicans* FLC resistant strain was isolated from the oral cavity of a pediatric patient infected with HIV and* C. albicans* (7173 serotype B) and was kindly provided by Hospital Evandro Chagas/FIOCRUZ/RJ. The microorganisms were maintained in Sabouraud dextrose agar for 48 h at room temperature.

### 2.4. Macrophage Culture

Macrophage cell line RAW 264.7 was purchased from the Rio de Janeiro cell bank. Cells were grown in Dulbecco's Modified Eagle Medium (DMEM) supplemented with 2 mM L-glutamine, 50 *μ*g/mL gentamicin, and 2.5 *μ*g/mL Fungizone*™*, plus 10% of heat-inactivated fetal bovine serum (FBS) and maintained at 37°C in an atmosphere of 5% CO_2_.

### 2.5. Evaluation of Inhibitory Concentrations

The minimum inhibitory concentration (MIC) was determined using the microdilution broth method according to CLSI M27-A [25]. First, the* O. basilicum* EO and linalool and geraniol standards were serially diluted into 96-well plates in duplicates. Then 100 *μ*L of cell suspension (10^3^ yeast/mL) was added to each well and the plate was incubated at room temperature for 24 or 48 h. Positive controls were prepared using yeast inoculated growth medium (untreated cells). Pure medium was used for the negative controls. Growth inhibition was confirmed after the addition of 30 *μ*L of resazurin solution (5 mg/100 mL of phosphate buffer saline, PBS; pH 7.2) and further incubation at 37°C for 3 h. FLC was used as the antimicrobial standard drug. MIC was defined as the lowest concentration of the EO, linalool, and geraniol that completely invalidated the microorganism growth.

### 2.6. Synergism Assay with the Antifungal Standard Drug FLC

The synergistic effect of the EO, linalool, and geraniol on FLC antifungal activity was performed as previously described by Zore et al. [23], with slight modifications. FLC and* O. basilicum* EO or its major components (linalool and geraniol) were combined at concentrations lower than their individual MIC values into 96-well microplates. Each plate was inoculated with 10^3^ cells/mL and incubated at room temperature for 24 h or 48 h, according to the requirements of the microorganisms. The results were based on visual growth, which were confirmed with the addition of resazurin as described above. Fractional inhibitory concentrations (FICs) for each substance and in their combination with FLC were calculated as follows:

(1) FICindex=concentration  that  inhibits  100%  of  growth  in  combinationconcentration  that  inhibits  100%  of  growth  alone.

The FIC_index_ was calculated by adding both FIC values. FIC_index_ values <0.5 and >4.0 have synergistic and antagonistic interactions, respectively [26].

### 2.7. Determination of the Amount of Ergosterol

Sterol extraction was performed as previously reported by Arthington-Skaggs et al. [27], with a slight modification. A single* C. albicans* or* C. neoformans* colony from an overnight Sabouraud dextrose agar plate culture was inoculated in 50 mL of Sabouraud dextrose broth containing different concentrations of the EO, linalool, and geraniol (625, 395, and 38 *μ*g/mL for* C. neoformans* and 625, 395, and 76 *μ*g/mL for* C. albicans*, resp.).* C. albicans* and* C. neoformans* cultures were incubated at 37°C for 18 and 24 h, respectively, with shaking. The cells were harvested by centrifugation and washed with sterile water. The wet weight of the cell pellet was determined. Three milliliters of 25% alcoholic potassium hydroxide solution (25 g of KOH and 36 mL of sterile distilled water, brought to 100 mL with 100% ethanol) was added and mixed by vortex for 1 min. Cell suspensions were incubated in a water bath at 85°C for 1 h. After the incubation period, the tubes were cooled at room temperature. The extraction of the sterols was carried out by the addition of 1 mL of sterile distilled water and 3 mL of cyclohexane and then mixing by vortex for 3 min. The cyclohexane layer was transferred to a clean borosilicate glass tube. Then, 200 *μ*L aliquot of sterol extract was diluted fivefold in 100% ethanol and scanned spectrophotometrically between 200 and 300 nm (DU 530 Life Science UV/Visible Spectrophotometer, Beckman Coulter) [27].

Both 24(28)-dehydroergosterol (24(28) DHE, a late sterol pathway intermediate) and ergosterol absorb at 281.5 nm, but only 24(28) DHE shows an absorption spectrum at 230 nm. Ergosterol content was calculated as a percentage of the wet weight of the cells using the following equations: % ergosterol + % 24(28) DHE = [(*A*_281.5_/290) × *F*]/pellet weight; % 24(28) DHE = [(*A*_230_/518) × *F*]/pellet weight, and % ergosterol = [% ergosterol + % 24(28) DHE]  −  % 24(28) DHE, where *F* is the factor for dilution in ethanol and 290 and 518 are the *E* values determined for crystalline ergosterol and 24(28) DHE, respectively [28].

### 2.8. Antibiofilm Formation Activity

The effects of the* O. basilicum* EO, linalool, and geraniol against* C. albicans* biofilm formation were evaluated. In order to assure better yeast adhesion, 96-well microplates were pretreated with 100 *μ*L of fetal bovine serum (FBS) for 30 minutes at 37°C. The microplates were washed with PBS and then 100 *μ*L of* C. albicans* suspension (1 × 10^7^ cells/mL) in YNB (yeast nitrogen base) broth, supplemented with 2% glucose, pH 7.0, was inoculated into the wells and incubated at 37°C for 90 min. Nonadherent cells were removed by washing the microplates twice with PBS, and then 100 *μ*L of different concentrations of the EO, linalool, and geraniol was added to the wells (final concentrations at one- and twofold MIC). After 48 h incubation at 35°C, the medium was removed and the biofilm was washed with PBS [29]. The untreated biofilms were used as positive control.

Biofilm quantification was made by the cellular reduction of salt XTT. First, 150 *μ*L of XTT-menadione solution (12.5 *μ*g/mL of XTT + 0.17 *μ*g/mL of menadione) in PBS was added to each well, and then the microplates were incubated for 2 h at 35°C in the dark. After the incubation period, 100 *μ*L from each well was transferred to another 96-well microplate and the optical density was determined at 475 nm (SpectraMax M5, Molecular Devices, USA) [29].

### 2.9. Transmission Electron Microscopy

*C. neoformans*, FLC sensitive, and resistant* C. albicans* strains were treated with subinhibitory concentrations of the EO (625 *μ*g/mL for all microorganisms) and geraniol (38 *μ*g/mL for* C. neoformans* and 76 *μ*g/mL for both* C. albicans* strains) for 48 h at 37°C. The yeasts were washed in PBS, pH 7.2, and fixed in a solution of 2.5% glutaraldehyde and 4% formaldehyde in 0.1 M cacodylate buffer, pH 7.2, for 1 h at room temperature. Next, the yeasts were postfixed for 2 h in 1% osmium tetroxide containing 1.25% potassium ferrocyanide and 5 mM CaCl_2_ in cacodylate buffer, pH 7.2. The yeasts were then washed in the same buffer, dehydrated with increasing ethanol concentrations (30, 50, 70, 90, and 100% and ultradry ethanol), with the cells remaining in each concentration for 30 min, and then embedded in Spurr resin. Ultrathin sections were stained with uranyl acetate and lead citrate, and images were obtained using a Zeiss 900 electron microscope equipped with a CCD Camera (mega view III model, Soft Image System, Germany). The images were processed with iTEM software (Soft Image System, Germany).

### 2.10. *C. neoformans* Capsule Size

*C. neoformans* was incubated for 48 h at 35°C in the presence of subinhibitory concentrations of EO, linalool, and geraniol (625, 395, and 38 *μ*g/mL, resp.). After 48 h, an aliquot was removed and fixed with a formaldehyde solution (10% formaldehyde in PBS) to measure capsule sizes. Slides were prepared for measurements with a drop of cells and a drop of India ink dye to enable capsule visualization. The images were obtained using a Zeiss Axioplan microscope. Measurements were performed using ImageJ software considering the distance between the cell wall and the outer edge of the capsule. Each cell was measured in four different regions to obtain the average capsule size. Thirty cells were analyzed for each condition.

### 2.11. Cytotoxicity

Cytotoxicity was assessed via MTT reduction as previously described [30]. Several concentrations of* O. basilicum* EO, linalool, and geraniol ranging from 5000 to 310 *μ*g/mL, 3156 to 197 *μ*g/mL, and 612 to 38.3 *μ*g/mL, respectively, were placed in contact with the RAW cell cultures and incubated at 37°C for 24 h. Next, 10 *μ*L of MTT solution was added to cell cultures and incubated for 3 h at 37°C. The formazan crystals formed were solubilized by adding 100 *μ*L of dimethylsulfoxide (DMSO). The absorbance was determined at 490 nm in an automatic spectrophotometer (ELx800TMBio-TeK Instruments, Inc.). The 50% cytotoxic concentration (CC50) was defined as the compound concentration which caused a 50% reduction in the number of viable cells.

## 3. Results and Discussion

The results of minimum inhibitory concentrations are shown in Table 1. The MIC values found for the EO against* C. neoformans*,* C. albicans sensitive,* and* C. albicans resistant strain* were similar (1250 *μ*g/mL). Geraniol was the most effective compound against all the strains tested (76 *μ*g/mL against* C. neoformans* and 152 *μ*g/mL against the two* C. albicans* strains tested). However, different results were observed with linalool. The MIC for linalool was 790 *μ*g/mL against* C. neoformans* and* C. albicans* sensitive and 1580 *μ*g/mL against the* C. albicans* fluconazole resistant strain. Our preliminary results with MIC corroborate with reports in the literature that show antimicrobial properties of extracts and essential oils of other natural products and their major components [31–37].

These results encouraged us to evaluate the combination of EO, linalool, and geraniol with a standard drug. Previous works have reported that the combination of different antifungal agents or the combination of natural products and standard drugs could reduce their separate MIC values. Liu et al. [38] showed that the combination of FLC and glabridin, an isoflavan isolated from* Glycyrrhiza glabra*, reduced the MIC values of* C. neoformans*, indicating a synergistic effect. Faria et al. [39] found a synergistic effect in the combination of 2,5-dihydroxybenzaldehyde and FLC against* C. neoformans*. In this work, we evaluate the effect of EO and its major components (geraniol and linalool) with the reference drug FLC and the combination of the two major components against* C. neoformans*. All the combinations tested produced FIC_index_ values ranging from 0.3826 to 0.6326. This showed that all these combinations reduced the MIC values. The synergistic effect was observed in the combination of FLC and geraniol, reducing their MIC from 31.25 to 4.14 *μ*g/mL and 76 to 19 *μ*g/mL, respectively, and in the combination of linalool with geraniol, reducing their MIC values from 790 to 111 *μ*g/mL and 76 to 19 *μ*g/mL, respectively. When FLC was combined with 197 *μ*g/mL linalool and 625 *μ*g/mL EO, their MIC values were reduced from 31.25 to 8.054 and 4.14 *μ*g/mL in the respective combinations (Table 1). It is important to note that, in combination, the needed concentrations of the two major components together to completely eradicate* C. neoformans* were very low. No combination of* O. basilicum* EO and commercial antifungal agents against* C. neoformans* was found in the literature consulted.

For the* Candida albicans* strains all the combinations tested produced FIC_index_ values ranging from 0.127 to 0.57. Although all the combinations reduced the MIC values in at least one of the paired substances, we did not observe any synergistic effect in the combinations of natural components with FLC against* C. albicans* sensitive. However, a synergistic effect was observed in the combination of linalool with geraniol, reducing their MIC values from 790 to 105 *μ*g/mL and 152 to 38 *μ*g/mL, respectively. Furthermore, all combinations tested presented synergistic effect against* C. albicans* resistant. When FLC was combined with EO 156 *μ*g/mL, its MIC value was reduced from 500 to 1.01 *μ*g/mL. The combination of FLC with 197 *μ*g/mL linalool and 38 *μ*g/mL geraniol reduced its MIC value from 500 to 2.02 *μ*g/mL and to 1.04 *μ*g/mL, respectively. This is a significant result because when the high concentrations of the standard drugs are reduced the collateral effects are also reduced. Moreover, the combination of linalool and geraniol caused complete cellular inhibition at reduced MICs of 4.8 *μ*g/mL and 397 *μ*g/mL for geraniol and linalool, respectively (Table 1). These results corroborate with previously reported results that show the synergistic effect of geraniol and FLC against* C. albicans* [22]. However, when compared with our study, a higher concentration of geraniol (140 *μ*g/mL) was necessary to reduce the MIC value of FLC from 64 to 2 *μ*g/mL.

**Figure 1:**
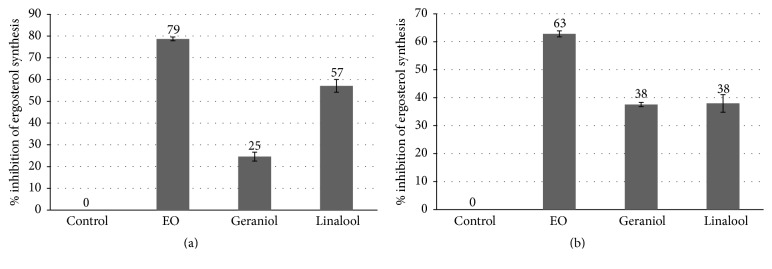
Effect of 625 *μ*g/mL of EO, 395 *μ*g/mL of linalool, and 38 *μ*g/mL of geraniol on the inhibition of ergosterol synthesis in* C. neoformans* (a) and the effect of 625 *μ*g/mL of EO, 395 *μ*g/mL of linalool, and 76 *μ*g/mL of geraniol on the* C. albicans* sensitive strain (b). The results represent the mean ± standard error of two independent experiments in triplicate.

In order to investigate the mechanisms of action of EO, linalool, and geraniol, we analyzed the inhibition of ergosterol synthesis. Ergosterol is an important sterol presented in the yeast cell membrane that controls membrane fluidity and integrity, and it is an important target for some antifungals [40–42]. The treatment of* C. neoformans* with EO (625 *μ*g/mL), linalool (395 *μ*g/mL), and geraniol (38 *μ*g/mL) results in an inhibition of 79, 57, and 25% of ergosterol synthesis, respectively (Figure 1(a)). Even in subinhibitory concentrations, the substances tested were able to reduce the ergosterol content. This is an indication that they might act in the ergosterol biosynthesis pathway, especially the EO and linalool.

The treatment of* C. albicans* sensitive with EO (625 *μ*g/mL), linalool (395 *μ*g/mL), and geraniol (76 *μ*g/mL) resulted in an inhibition of 63, 38, and 38% of ergosterol synthesis, respectively. A similar effect was obtained with* C. albicans* resistant (Figure 1(b)). These data corroborate with those reported by Khan et al. In that study, the treatment with* O. sanctum* essential oil and linalool was also able to decrease ergosterol synthesis in* C. albicans* [43]. However, in another study, geraniol and linalool were not effective in reducing the ergosterol content in* C. albicans*, even at higher concentrations (4 mg/mL and 8 mg/mL, resp.) [44].

**Figure 2:**
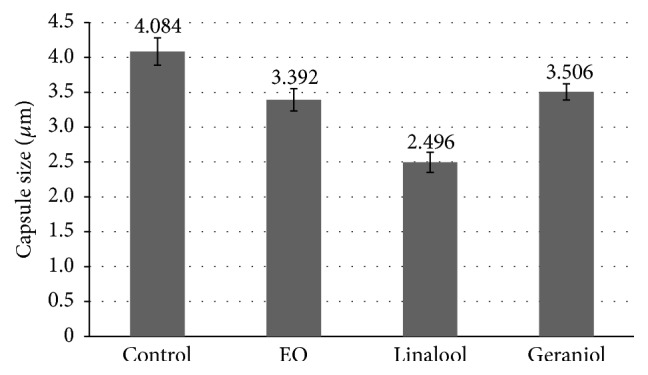
Effect of 625 *μ*g/mL EO, 395 *μ*g/mL linalool, and 38 *μ*g/mL geraniol on the capsule size.

Another mechanism of action analyzed in this study was the ability of the studied components to reduce the capsule size of* C. neoformans*. The capsule is the major virulence factor of* C. neoformans* and it is an important target of study [45]. In the sub-MIC concentrations tested, linalool showed the highest activity in reducing the capsule size, followed by EO, and geraniol was the least active (Figure 2). The presence of capsules can alter the susceptibility of antimicrobial drugs; therefore, finding natural products that reduce capsule size is extremely important. The results here suggest that the three substances tested can reduce the capsule size; and therefore they might be used with other antimicrobial drugs to reduce the concentration needed and consequently its collateral effects. Vitale et al. tested the activity of antifungal agents against strains of* C. neoformans* with different capsule sizes. They showed that the presence of capsules affects the susceptibility of the yeast to the antimicrobial tested [46].

**Figure 3:**
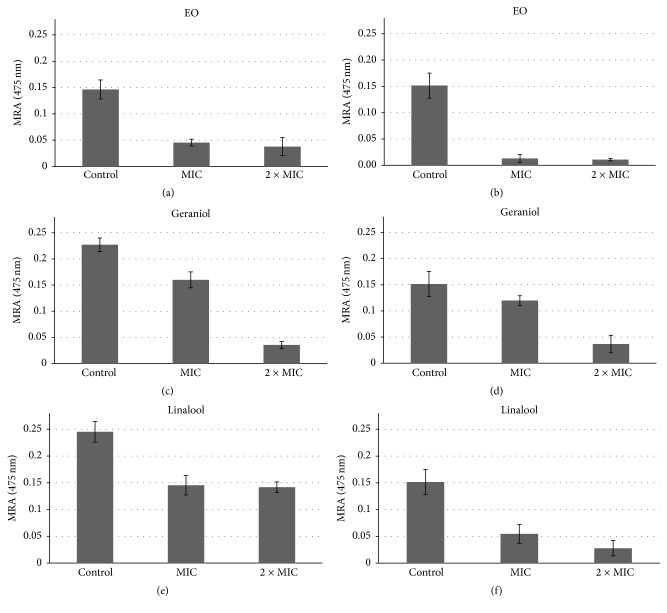
Effect of* O. basilicum* EO, geraniol, and linalool standards on the mitochondrial reducing activity (MRA) of biofilms formed by* C. albicans* sensitive (a, c, e) and* C. albicans* fluconazole resistant (b, d, and f) treated with one- and twofold MIC concentrations. The results represent the mean ± standard error of two independent experiments in triplicate. Values over the bars refer to the percentage of inhibition of biofilm viability.

The activity of the EO, linalool, and geraniol against* C. albicans* biofilm formation was evaluated and the EO was the most effective. At 2x, its MIC value geraniol appears to be more effective against* C. albicans* sensitive biofilm formation than* C. albicans* resistant. EO showed the highest biofilm inhibitory activity against both* C. albicans* strains at MIC followed by linalool and geraniol, respectively (Figure 3). Alviano et al. demonstrated that the essential oil of* Croton cajucara* and purified linalool were effective against artificial biofilms of* C. albicans *[47]. A previous study showed that the essential oil of* Boesenbergia pandurata*, with a high content of geraniol (56.68%) and 2.36% of linalool, was able to interfere in the initial phases of* Candida* biofilm formation [48]. Another study showed a strong antimicrobial activity of the essential oil of* O. americanum* L. against the planktonic form of* C. albicans* but a less pronounced effect on these microorganisms in biofilms [49]. The biofilm inhibitory activities of linalool and geraniol obtained in the present study are consistent with those described by Dalleau et al., who demonstrated that geraniol was the most effective substance against* C. albicans* biofilm [50].

**Figure 4:**
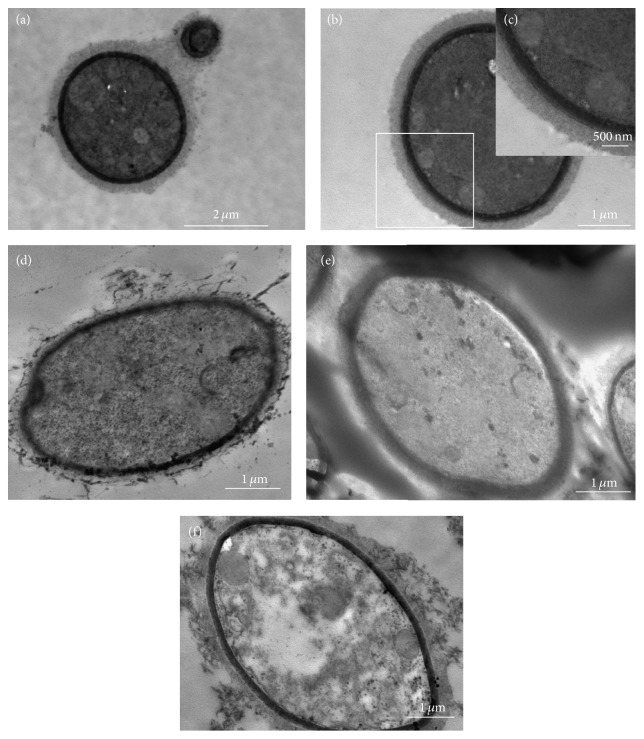
Transmission electron microscopy of* C. neoformans* T444 strain treated with 625 *μ*g/mL of EO, 38 *μ*g/mL of geraniol, and 395 *μ*g/mL of linalool for 48 h at 37°C. Untreated cells (control) showed good preservation of cell wall and membrane and the final of budding (a–c). The cells treated with 625 *μ*g/mL of EO (d), 38 *μ*g/mL of geraniol, (e) and 395 *μ*g/mL of linalool (f) showed a disruption of the fungal capsule structure.

In order to observe the ultrastructural changes in the* C. neoformans* strain after treatment with EO, linalool, and geraniol, the treated and untreated cells, grown for 48 h at 37°C, were processed for transmission electron microscopy. Untreated cells (control) showed good preservation of cell walls and membrane and the final of budding (Figures 4(a)–4(c)). The cells treated with 625 *μ*g/mL of EO (Figure 4(d)), 38 *μ*g/mL of geraniol (Figure 4(e)), and 395 *μ*g/mL of linalool (Figure 4(f)) showed a disruption of the fungal capsule structure.

**Figure 5:**
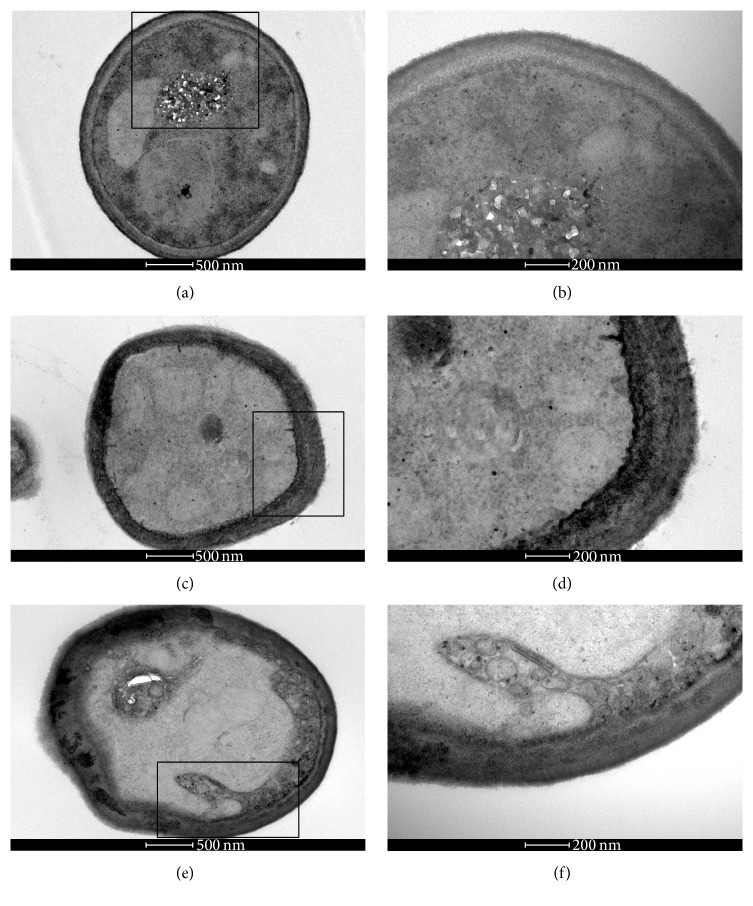
Transmission electron microscopy of* C. albicans* fluconazole sensitive strain treated with 625 *μ*g/mL of EO and 76 *μ*g/mL of geraniol for 48 h at 37°C. The control (untreated)* C. albicans* sensitive strain cells showed good preservation of cell wall and membrane (a and b). Cells treated with 625 *μ*g/mL of EO presented cell wall thickening (c and d) and the treatment with 76 *μ*g/mL of geraniol led to the appearance of cell membrane invaginations presenting some vesicles and the thickening of the cell wall (e and f).

**Figure 6:**
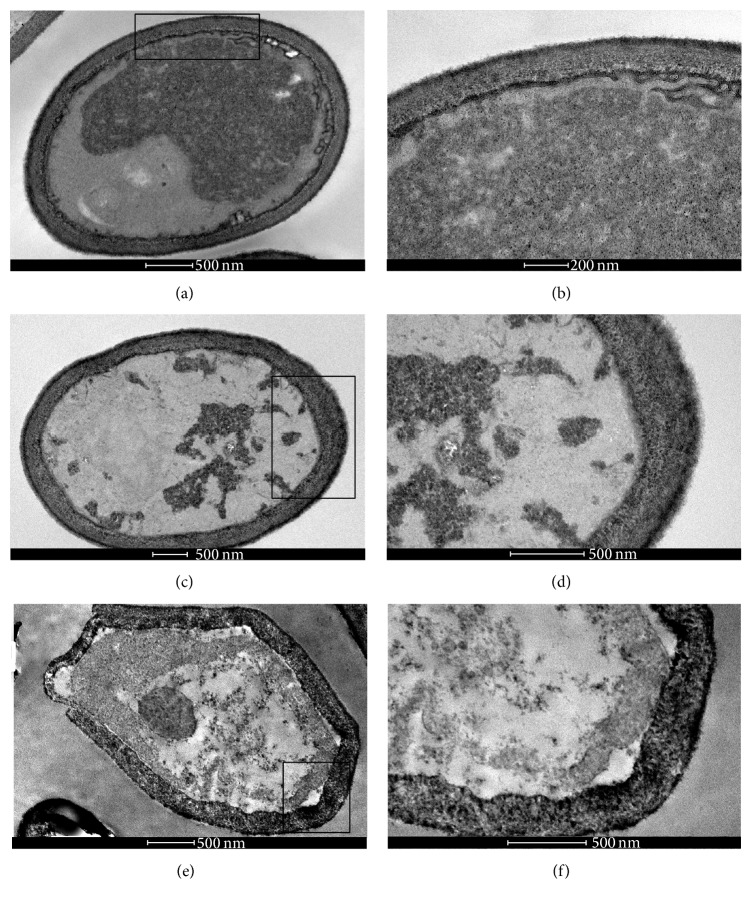
Transmission electron microscopy of* C. albicans* fluconazole resistant strain treated with 625 *μ*g/mL of EO and 76 *μ*g/mL of geraniol for 48 h at 37°C. Control cells showed good preservation of the membrane, cell wall, and small vesicles near the membrane (a and b). Yeasts treated with subinhibitory concentrations of the essential oil and geraniol showed cell wall thickening and irregularities in the membrane (c and d). Cells treated with subinhibitory concentration of geraniol presented cell wall thickening and irregularities in cell membrane, suggesting budding failure (e and f).

The control (untreated)* C. albicans* sensitive strain cells showed good preservation of their cell walls and membranes (Figures 5(a) and 5(b)). Cells treated with 625 *μ*g/mL of EO presented cell wall thickening (Figures 5(c) and 5(d)) and the treatment with 76 *μ*g/mL of geraniol led to the appearance of cell membrane invaginations presenting some vesicles and the thickening of the cell walls (Figures 5(e) and 5(f)). The control cells of* C. albicans* fluconazole resistant strain showed good preservation of the membrane, cell wall, and small vesicles near the membrane (Figures 6(a) and 6(b)). However, yeasts treated with subinhibitory concentrations of EO and geraniol showed cell wall thickening and irregularities in the membrane (Figures 6(c) and 6(d)). Cells treated with a subinhibitory concentration of geraniol presented cell wall thickening and irregularities in the cell membrane, suggesting budding failure (Figures 6(e) and 6(f)).

**Figure 7:**
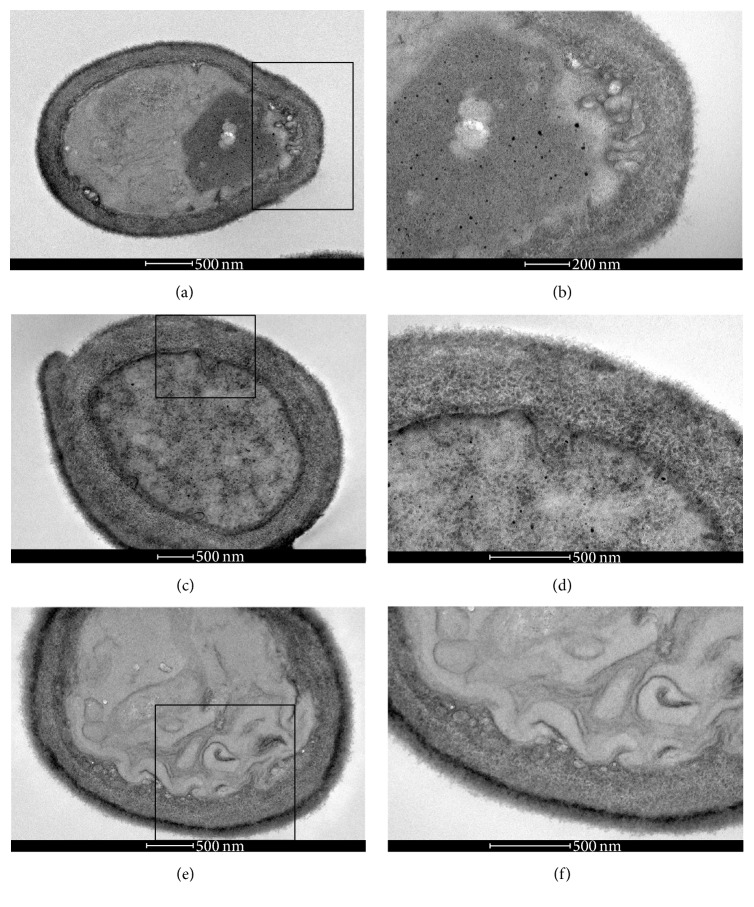
Transmission electron microscopy of* C. albicans* fluconazole resistant strain treated with FLC alone (1 *μ*g/mL) and combined with geraniol (38 *μ*g/mL) and EO (156 *μ*g/mL); the latter two trails with FLC exhibited synergistic activity. The treatment with 1 *μ*g/mL of FLC showed cells with irregularity in their membranes and formation of small vesicles in the proximity (a-b). In the treatment with the combination of essential oil 156 *μ*g/mL and FLC 1 *μ*g/mL, the cells demonstrated a very thick cell wall and membrane invaginations (c-d). Cells treated with the combination of geraniol 38 *μ*g/mL and FLC 1 *μ*g/mL showed extensive irregularities in the membrane and formation of membrane vesicles in the cytoplasm (e-f).

In Figure 7, the transmission electron microscopy of* C. albicans* fluconazole resistant strain treated with FLC alone and in combination with geraniol and EO exhibited a synergistic activity with FLC. FLC treated with 1 *μ*g/mL cells has irregularity in the membrane and formation of small vesicles in the proximity (Figures 7(a) and 7(b)). Treated with the combination of essential oil 156 *μ*g/mL and FLU 1 *μ*g/mL cells presented a very thick cell wall and membrane invaginations (Figures 7(c) and 7(d)). Cells treated with the combination of geraniol 38 *μ*g/mL and FLU 1 *μ*g/mL showed large irregularities in the membrane and formation of membrane vesicles in the cytoplasm (Figures 7(e) and 7(f)).

The cytotoxic activities of essential oil, geraniol, and linalool were analyzed on a RAW cell line and showed CC_50_ at a concentration of 380 *μ*g/mL for geraniol and less than 310 *μ*g/mL and 197 *μ*g/mL (the lowest concentrations evaluated) for essential oil and linalool, respectively. According to this, the MIC values found for geraniol were much lower than the CC_50_. Using the checkerboard, MIC values of linalool and EO were reduced to 105 and 156 *μ*g/mL, respectively, in some combinations, which can minimize the cytotoxic activity of these components.

## 4. Conclusions

In this study we showed that geraniol displayed the highest activity against* C. neoformans* and the strains of* C. albicans* tested and the lowest cytotoxicity, when compared with linalool and the EO The ergosterol inhibition and the synergism showed the potential activity of this natural product and that its combination with standard drugs can be useful against the microorganisms tested. The anticryptococcal activity described here encourages the search for more effective substances of vegetal origin for the treatment of cryptococcosis.

Besides the antifungal activity described here the results support the use of* O. basilicum* essential oil as a folk medicine and open perspectives to find more effective substances from vegetal origin for the treatment of fungal diseases.

## Figures and Tables

**Table 1:** Evaluation of interaction resulting from the combination of the substances tested by determining the FIC_index_ using the checkerboard technique.

Strain	MIC (*µ*g/mL)				MIC in combination (*µ*g/mL)											
	EO	L^a^	G^b^	FLC^c^	FLC	EO	FIC^d^ index	FLC	L	FIC index	FLC	G	FIC index	L	G	FIC index
*C. neoformans*	1250	790	76	31.25	4.14	625	0.633 (I)^e^	8.054	197	0.5077 (I)	4.14	19	0.3826 (S)	111	19	0.3905 (S)
*C. albicans resistant*	1250	1580	152	500	1.01	156	0.127 (S)^f^	2.02	197	0.134 (S)	1.04	38	0.252 (S)	397	4.8	0.284 (S)
*C. albicans sensitive*	1250	790	152	0.975	0.259	312	0.51 (I)	0.065	395	0.57 (I)	0.259	38	0.51 (I)	105	38	0.38 (S)

^a^L: linalool; ^b^G: geraniol; ^c^FLC: fluconazole;  ^d^FIC index: fractional inhibitory concentration index; ^e^I: indifferent; ^f^S: synergist.

## Appendix

See Table 1.
